# Longitudinal associations between energy utilization and brain volumes in cognitively normal middle aged and older adults

**DOI:** 10.1038/s41598-022-10421-7

**Published:** 2022-04-19

**Authors:** Yujia Qiao, Amal A. Wanigatunga, Yang An, Fangyu Liu, Adam P. Spira, Christos Davatzikos, Qu Tian, Eleanor M. Simonsick, Luigi Ferrucci, Susan M. Resnick, Jennifer A. Schrack

**Affiliations:** 1 Department of Epidemiology, Johns Hopkins Bloomberg School of Public Health, Baltimore, MD USA; 2 Center on Aging and Health, Johns Hopkins University, Baltimore, MD USA; 3 Intramural Research Program, National Institute on Aging, Baltimore, MD USA; 4 Department of Mental Health, Johns Hopkins Bloomberg School of Public Health, Baltimore, MD USA; 5 Department of Psychiatry and Behavioral Sciences, Johns Hopkins School of Medicine, Baltimore, MD USA; 6 Department of Radiology, University of Pennsylvania, Philadelphia, PA USA

**Keywords:** Diseases, Risk factors, Signs and symptoms

## Abstract

Peak energy capacity of the whole person is associated with neurodegeneration. However, change in ability to utilize energy manifests as combination of declining peak energy capacity and rising energetic costs of mobility in mid-to-late life. We examined longitudinal associations between change in energy utilization and brain volumes. Cognitively normal participants from the Baltimore Longitudinal Study of Aging (N = 703, age = 70.4 ± 12.1 years, 54.1% women, 30% black) had concurrent data on brain volumes and energy utilization (defined as ratio of energetic cost of walking to peak energy capacity (“cost-to-capacity ratio”) at ≥ 1 visit between 2008 and 2018. We performed linear mixed-effect models, adjusting for demographics, medical history and walking engagement. Average baseline cost-to-capacity ratio was 0.55 ± 0.16, with average annual increase of 0.04 ± 0.13 over 3.9 follow-up years. A 10% higher baseline cost-to-capacity ratio was associated with 2.00 cm^3^ (SE = 0.44) larger baseline ventricular volume (p < 0.001), and 0.10 cm^3^ (SE = 0.03) greater annual increase in ventricular volume (p = 0.004) after adjustment. Longitudinal change in cost-to-capacity ratio was not associated with brain volumes. These findings highlight, among cognitive-normal adults, poorer baseline energy utilization is associated with subsequent ventricular enlargement, an indirect measure of central brain atrophy. Future studies should explore whether early detection of worsening energy utilization may act as a marker of underlying brain atrophy.

## Introduction

The brain accounts for approximately 20% of total daily energy expenditure in humans^1^, yet little is known about the association between efficiency of energy utilization and brain health. To date, research has primarily focused on the construct of peak energy capacity (e.g., peak/max VO_2_) on cognitive performance and brain health^2–7^. However, beginning in mid-life, changes in the ability to utilize energy manifest as a combination of declining peak capacity and rising energetic costs of mobility^8^. These additional energetic costs are important, particularly as they expand to consume a larger portion of peak energy availability, leaving less energy for daily activities and leading to higher fatigability^9,10^. Many underlying mechanisms contribute to these rising energetic costs, including age- and disease-related alterations in metabolism, biomechanics, and neuromotor control^11,12^, which may further reduce energy availability. Over time, this “compression” of energy reserves pushes the cost of mobility beyond 50% of peak energy capacity, often approaching 100% of peak energy capacity in old age^12–14^. Previous work by our group has shown that compromised energy utilization is associated with slow and declining gait speed, greater fatigability, and faster decline in memory with aging^12–14^, but its association with concurrent brain structure and changes in brain structure has not been explored.

Previous neuroimaging studies among mid-to-late life populations, relying largely on cross-sectional design, suggest some associations between peak energy capacity and brain volumes but findings are inconsistent^2–5^. Specifically, a meta-analysis of 11 cross-sectional studies found a positive association between VO_2max_ and white matter volume^5^; yet another study of middle-to-older aged adults found no association with white matter volume, but a significant positive association between VO_2max_ and gray matter volume^2^. Importantly, the associations between peak energy capacity and brain volumes may differ by cognitive status; a study of older adults aged ≥ 60 years found VO_2peak_, an approximation of VO_2max_, was associated with whole brain and white matter volumes in participants with early Alzheimer’s disease, but not in nondemented participants^3^. Moreover, the cross-sectional nature of these works limits inferences in temporality.

Although limited longitudinal work suggests peak energy capacity is associated with brain volumes in selected middle and medial regions, including the parahippocampal gyrus^6,7^. Focusing on peak energy capacity alone may not sufficiently inform the effects of daily energy availability on brain structure, particularly among those whose energy demands for mobility exceed 50% of the peak energy capacity^15,16^. Accordingly, we focused on energy utilization, which simultaneously examines the age-associated upward trend in energetic costs for mobility in relation to the downward trend in peak energy expenditure^14^, and examined the magnitude of the cross-sectional and longitudinal associations between energy utilization and brain volumes in cognitively normal participants from the Baltimore Longitudinal Study of Aging (BLSA). We hypothesized that those with poorer energy utilization at baseline, or greater declines in energy utilization, would exhibit greater brain atrophy over time.

## Methods

### Study participants

The BLSA is a study of human aging established in 1958 and conducted by the National Institute on Aging Intramural Research Program. The BLSA consists of a continuously enrolled cohort of community dwelling volunteers who pass comprehensive health and functional screening evaluations and are free of major chronic conditions, except controlled hypertension, and cognitive and functional impairment at the time of enrollment. Once enrolled, participants are followed for life or until a diagnosis of dementia and undergo extensive testing every 1–4 years depending on age (< 60 every 4 years, 60–79 every 2 years, ≥ 80 every year)^8^.

A sub-sample of the BLSA of around 1000 participants has been prospectively assessed using magnetic resonance imaging (MRI) to measure global and regional brain structure. The current study sample consisted of all participants ≥ 50 years old with brain imaging, energy assessments, and body composition measures (N = 756). For each participant, the first visit with all assessments served as baseline. Visits were excluded if participants had missing data on any global or lobar brain regions (n = 18), body composition (n = 25), education (n = 2), or Center for Epidemiologic Studies Depression Scale (CES-D) score (n = 9). Participants were also excluded from the analysis after the onset of mild cognitive impairment (MCI) or Alzheimer’s disease, and all included participants reported no history of Parkinson’s disease. Comparison of baseline characteristics between those included and excluded did not reveal significant differences (Supplementary Table 1). The final sample included 703 participants aged 50–94 years assessed between 2008 and 2018. The study protocol was approved by the Institutional Review Board of the Intramural Research Program of the National Institutes of Health, and all methods were performed in accordance with the relevant guidelines and regulations. All participants provided written informed consent at each visit.

### Energy utilization (cost-to-capacity ratio)

Average peak walking capacity VO_2_ (ml/kg/min) was assessed during the 400-m segment of the long-distance corridor walk; a rapid-paced endurance walking test and a validated proxy of cardiorespiratory fitness in older adults^17^. The test was performed on a 20-m course in an uncarpeted corridor marked by cones at both ends, with the participant wearing a portable metabolic analyzer, the Cosmed K_4_b^2^ (Cosmed, Rome, Italy). Participants were instructed to walk “as fast as possible, at a pace you can sustain for 400 m”. Standardized encouragement was given with each lap along with the number of laps remaining. Split times for each lap and total time to walk 400 m were recorded. To calculate average peak walking energy expenditure per kilogram of body weight (peak VO_2_ ml/kg/min), readings from the first 1.5 min of the test were discarded to allow the participant to adjust to the peak workload and the remaining readings were averaged to arrive at single measure of the average energy expended (ml/kg/min) during 400 m of peak sustained walking^11,14^.

The energetic cost of slow walking (ml/kg/min) was assessed via indirect calorimetry (Medical Graphics Corp, St Paul, MN) during 5 min of treadmill walking at 0.67 m/s (1.5 mph), 0% grade. This constant speed and duration were used for all participants, providing a standardized measure by which to gauge changes in energy expenditure (e.g., walking efficiency) during a low-demand task that minimizes exclusion of lower functioning participants^11,14^. To calculate the average volume of oxygen consumed per kilogram of body weight (ml/kg/min) during the walking task, energy expenditure readings from the first 2 min of testing were discarded to allow the participant to adjust to the workload. The final 3 min were averaged to derive a single measure of the average VO_2_ (ml/kg/min) consumed, or the average energetic cost of a slow standardized walking task^11,14^.

A ratio of the energetic cost of walking to peak walking energy expenditure (the “cost-to-capacity ratio”)^11,14^ was calculated to define the percentage of peak walking capacity needed for mobility:

$$\frac{{{\text{Energetic}}\,{\text{cost}}\,{\text{of}}\,{\text{slow}}\,{\text{walking }}\left( {{\text{ml}}/{\text{kg}}/{\text{min}}} \right)}}{{{\text{Peak}}\,{\text{walking}}\,{\text{energy}}\,{\text{expenditure}}\left( {{\text{ml}}/{\text{kg}}/{\text{min}}} \right)}}.$$

The cost-to-capacity ratio is bounded at 0 and 1, with a higher ratio indicating less efficient energy utilization (i.e., poorer energy utilization).

### MRI acquisition

Scanning was performed on a 3 T Philips Achieva at the National Institute on Aging (NIA) Clinical Research Unit. T1-weighted magnetization-prepared rapid gradient echo (MPRAGE) scans were acquired (repetition time = 6.8 ms, echo time = 3.1 ms, flip angle = 8°, image matrix = 256 × 256, 170 slices, pixel size = 1 × 1 mm, slice thickness = 1.0 mm, field-of-view = 240 mm^2^). For regional labeling and volumetric analysis, a standardized procedure was applied to T1-weighted volumetric scans to align images in parallel with the anterior–posterior commissure place, remove extracranial tissue, and define regional labels for gray matter, white matter, and cerebrospinal fluid (CSF)^18,19^. The intracranial volume (ICV) was estimated for the baseline scan of each individual and used as a covariate in statistical analyses.

Regions of interest (ROIs) included total brain, gray matter, white matter, ventricles, frontal lobe, frontal gray matter, frontal white matter, temporal lobe, temporal gray matter, temporal white matter, parietal lobe, parietal gray matter, parietal white matter, occipital lobe, occipital gray matter, occipital white matter, and hippocampus. The volumes of ROIs (cm^3^) measured at each visit were treated as dependent variables in separate models.

### Covariates

Demographic and lifestyle characteristics, including baseline age, sex, race (white, non-white), years of education, smoking status (never/former smoker, current smoker), and alcohol consumption (> 7 vs. ≤ 7 drinks per week) were obtained via an interviewer-administered questionnaire. During the interview, participants were also asked to report whether they were ever told by a doctor or other health professional that they had any of the following conditions: diagnosed cardiovascular diseases including heart attack, angina, myocardial infarction, congestive heart failure, peripheral arterial disease, and vascular-related procedures (including coronary artery bypass grafting or angioplasty); stroke; hypertension or high blood pressure; high cholesterol; chronic obstructive pulmonary disease; diabetes mellitus; cancer; or osteoarthritis. A morbidity index was created using a binary variable, defined as having 0–1 or 2 or more conditions mentioned above. Self-reported engagement in volitional walking was also recorded in the question “did you do brisk walking in the past 2 weeks” (yes/no).

Weight and height were assessed in light clothing according to standard protocols. Total body dual-energy X-ray absorptiometry (DEXA) was performed using a Prodigy Scanner (GE, Madison, WI) and analyzed with version 10.51.006 software to obtain fat mass and lean mass. Depressive symptoms were measured by CES-D^20^. Apolipoprotein E (APOE) e4 carrier status (≥ 1 vs. 0 e4 alleles) was determined from DNA samples^21^. All covariates were treated as time-fixed at baseline.

Diagnostic status (normal, MCI/dementia) was determined by standard procedures detailed previously^22^. Participants meeting pre-specified criteria were reviewed at a research diagnostic case conference. Although participants were excluded from the analyses after the onset of mild cognitive impairment (MCI) or Alzheimer’s disease, they were still followed-up and we were able to determine their latest diagnostic status.

### Statistical analysis

Baseline participant characteristics were compared by the baseline cost-to-capacity ratio (≤ 0.50 vs > 0.50) using t-test for continuous variables and χ^2^ tests for categorical variables and were reported as mean ± standard deviation (SD) or frequencies (percentages). Alpha was set to 0.01 for ROIs analyses after correction of multiple comparison using false discovery rate (FDR). All analyses were performed using Stata version 16 (Statacorp, College Station, TX).

To quantify trajectories of changes in brain volumes in the overall sample, linear mixed-effect models with restricted maximum likelihood estimation were used. This approach can combine cross-sectional and longitudinal data, as it borrows information and improves the precision for cross-sectional associations^23^. Time was modeled as a continuous variable scaled to years since baseline. The fixed effects of interest consisted of baseline cost-to-capacity ratio, change in cost-to-capacity ratio from baseline, time, all covariates, and two-way interactions between baseline cost-to-capacity ratio and change in cost-to-capacity ratio with time. Change in cost-to-capacity ratio was calculated as cost-to-capacity ratio measured at the current visit minus the baseline cost-to-capacity ratio, and it was modeled as a time-varying predictor. Unstructured variances and covariance for random incepts and slopes were used. Test for multi-collinearity did not suggest collinearity among predictors (all VIF < 3). We also further explored the independent associations between energetic cost of slow walking and peak walking energy expenditure, after adjusting for each other, with brain volumes using the same linear mixed-effect model approach.

To address potential confounding, Model 1 was adjusted for ICV, the latest diagnostic status (subsequently impaired vs. remain normal), baseline age, sex, race, and years of education. Model 2 added height, fat mass, and lean mass to the covariates from Model 1, and Model 3 further adjusted for morbidity index, CES-D scores, APOE e4 allele status and walking engagement. Several sensitivity analyses were also conducted to further test the robustness of our results: (1) restricting the main analyses to a sub-population who were never diagnosed with MCI or dementia over extended follow-up; (2) adding interaction terms to assess the potential for effect modification by MCI/dementia diagnosis or APOE status (cost-to-capacity × subsequent normal/MCI/dementia status and cost-to-capacity × APOE status), and (3) stratifying participants based on their baseline energy utilization at a cost-to-capacity ratio of ≤ 0.50 vs > 0.50^14^.

## Results

### Participant characteristics

Of the overall sample with a mean age of 70.4 ± 12.2 years, over half (54.1%) were women, and around two thirds (70.0%) were of white race. The percentages of current smokers (1.7%) and heavy drinkers (16.9%) were low (Table 1). More than half reported a diagnosis of high cholesterol (59.9%), osteoarthritis (54.6%) and/or hypertension (52.2%), and the majority of participants (70.0%) had more than two morbidities. The average baseline cost-to-capacity ratio was 0.55 ± 0.16, meaning on average participants used approximately 55% of their energy capacity to walk at a slow pace (Table 1). On average, those with baseline cost-to-capacity ratio > 0.5 (n = 402, mean = 0.65 ± 0.12) tended to be older and less physical active. In addition, those with the higher baseline cost-to-capacity ratio had greater fat mass, lower lean mass, more adverse health conditions (e.g., hypertension, osteoarthritis, CVD, stroke, and more than two morbidities), higher depressive symptoms, and a higher proportion of subsequent MCI/dementia diagnosis. The average time since baseline for the overall sample (N = 703) was 2.2 ± 2.4 years, and among those with > 1 visits (n = 399) the average follow-up time was 3.9 ± 2.0 years (Table 1).

**Table 1:** Baseline characteristics of 703 BLSA participants by baseline cost-to-capacity ratio (≤ 0.5 vs. > 0.5), defined as the energy cost of slow walking to peak walking energy expenditure.

	Overall N = 703	Baseline cost-to-capacity ratio ≤ 0.5 n = 301	Baseline cost-to-capacity ratio > 0.5 n = 402	p value
Age (years)	70.4 ± 12.2	66.1 ± 13.7	73.7 ± 9.8	< 0.001
Women	380 (54.1)	161 (53.5)	219 (54.5)	0.795
White	492 (70.0)	218 (72.4)	274 (68.2)	0.222
Education (years)	17.0 ± 2.7	17.2 ± 2.5	16.9 ± 2.9	0.255
Height, in cm	167.6 ± 9.1	168.4 ± 8.9	167.0 ± 9.3	0.042
Fat mass (kg)	26.4 ± 9.6	24.8 ± 8.9	27.6 ± 10.0	< 0.001
Lean mass (kg)	46.4 ± 9.8	47.3 ± 10.3	45.7 ± 9.3	0.028
Current smoker	12 (1.7)	5 (1.66)	7 (1.74)	0.935
Heavy drinker^a^	119 (16.9)	52 (17.28)	67 (16.67)	0.831
**Having more than 2 morbidities**	492 (70.0)	198 (65.8)	294 (73.1)	0.035
Having high cholesterol	421 (59.9)	174 (57.8)	247 (61.4)	0.330
Having osteoarthritis	384 (54.6)	150 (49.8)	234 (58.2)	0.027
Having hypertension^b^	367 (52.2)	133 (44.19)	234 (58.21)	< 0.001
Having cancer	217 (30.9)	86 (28.6)	131 (32.6)	0.254
Having diabetes^c^	111 (15.8)	43 (14.29)	68 (16.92)	0.344
Having COPD	95 (13.5)	38 (12.6)	57 (14.2)	0.551
Having CVD^d^	60 (8.5)	16 (5.3)	44 (11.0)	0.008
Having stroke	28 (4.0)	6 (2.0)	22 (5.5)	0.020
CES-D (range 0–60)	4.8 ± 5.1	4.3 ± 4.5	5.2 ± 5.4	0.013
APOE e4 carriers	173 (25.7)	84 (29.07)	89 (23.12)	0.080
Did brisk walking in the past 2 weeks	264 (37.6)	136 (45.2)	128 (31.8)	< 0.001
Intracranial volume (cm^3^)	1391.6 ± 140.8	1395.1 ± 144.5	1389.0 ± 138.0	0.571
Subsequent diagnosed MCI/dementia	84 (11.9)	27(8.97)	57(14.18)	0.035
Follow-up time (years)	3.9 ± 2.0	4.4 ± 2.0	3.6 ± 1.9	0.003
**Number of participants by visit**				0.656
1	304 (43.2)	127 (42.2)	177 (44.0)	
2	209 (29.7)	87 (28.9)	122 (30.4)	
3	123 (17.5)	54 (17.9)	69 (17.2)	
4+	67 (9.5)	33 (11.0)	34 (8.5)	
Baseline cost-to-capacity ratio	0.55 ± 0.16	0.40 ± 0.09	0.65 ± 0.12	< 0.001
**Baseline brain volumes (cm**^**3**^**)**				
Total brain	1134.3 ± 115.3	1146.9 ± 117.6	1124.9 ± 112.7	0.012
Gray matter	611.7 ± 61.9	622.5 ± 62.9	603.6 ± 60.0	< 0.001
White matter	465.5 ± 50.7	472.5 ± 52.8	460.3 ± 48.4	0.002
Ventricles	36.2 ± 22.5	30.8 ± 16.7	40.3 ± 25.3	< 0.001
Frontal lobe	350.0 ± 38.6	356.0 ± 39.4	345.4 ± 37.4	< 0.001
Temporal lobe	201.1 ± 22.4	204.1 ± 23.4	198.9 ± 21.5	0.002
Parietal lobe	172.9 ± 18.8	176.0 ± 19.6	170.7 ± 17.9	< 0.001
Occipital lobe	114.7 ± 13.9	116.8 ± 14.3	113.1 ± 13.5	0.001
Hippocampus	7.4 ± 0.8	7.5 ± 0.8	7.3 ± 0.9	0.003

All reported in mean ± SD or frequency (%). *SD* standard deviation, *COPD* chronic obstructive pulmonary disease, *CVD* cardiovascular disease, *CES-D* Center for Epidemiologic Studies Depression Scale, *MCI* mild cognitive impair.

^a^Drinking more than 7 alcoholic drinks/week.

^b^Systolic blood pressure ≥ 130 mmHg and or diastolic blood pressure ≥ 80 mmHg, or a history of diagnosis plus treatment with antihypertensive medications.

^c^Fasting glucose ≥ 126 mg/dL, or a history of a diagnosis plus treatment with oral antidiabetic drugs or insulin.

^d^CVD includes heart attack, angina, myocardial infarction, congestive heart failure, peripheral arterial disease, and vascular-related procedures (including coronary artery bypass grafting or angioplasty).

### Energy utilization-related brain volumetric change

**Figure 1:**
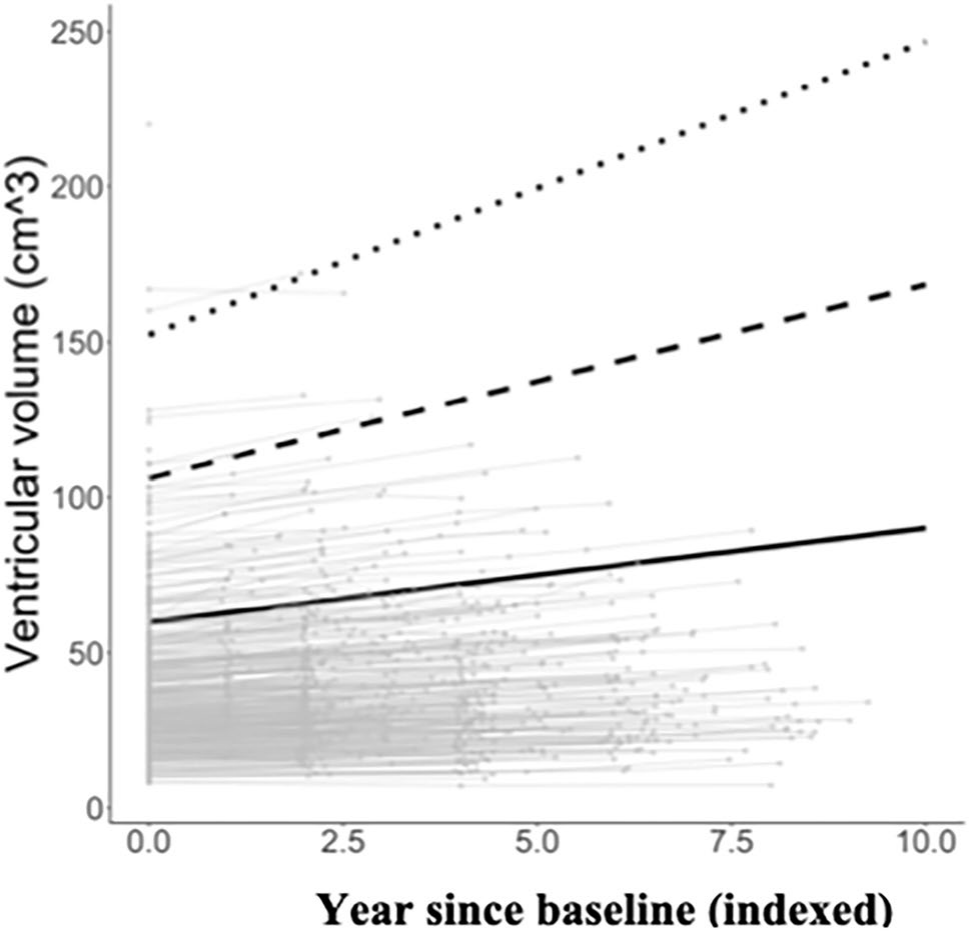
Adjusted baseline and annual change of ventricular volume at years since baseline (index). Solid black lines represent the estimated average if baseline cost-to-capacity ratio = 0.2 (good energy utilization), dashed black lines represent the estimated average volume if baseline cost-to-capacity ratio = 0.5 (moderate energy utilization), and dotted black lines represent the estimated average volume if baseline cost-to-capacity ratio = 0.8 (poor energy utilization). To better see individual trajectories, gray dots and gray lines at the background represent crude individual data points in this study. All estimated averages were fitted using linear mixed effect models after adjustment^a^. ^a^ Model (model 3) adjusted for ICV, the latest diagnostic status, baseline age, sex, race, years of education, height, fat mass, lean mass, number of multi-morbidities, CES-D, APOE e4 status and brisk walking.

In adjusted models (Model 3), a 10% higher baseline cost-to-capacity ratio was significantly associated with 2.0 cm^3^ (SE = 0.44, *p* < 0.001) larger ventricular volume at baseline (Table 2, Column A), and a 0.10 cm^3^ (SE = 0.03, *p* = 0.004) greater annual increase in ventricular volume (Table 2, Column B). Participants with poorer baseline energy utilization appeared to have a steeper annual change in ventricular volume (Fig. 1), but there were no significant longitudinal associations (Table 2, Column C, D).

**Table 2:** Longitudinal mixed models of the associations between energy utilization and brain volumes among 703 BLSA participants^a^.

	Baseline cost-to-capacity ratio (A)		Baseline cost-to-capacity ratio × time (B)		Change in cost-to-capacity ratio (C)		Change in cost-to-capacity ratio × time (D)		Time (E)	
	β (SE)	p-value	β (SE)	p-value	β (SE)	p-value	β (SE)	p-value	β (SE)	p-value
Total brain	0.260 (1.019)	0.799	0.187 (0.167)	0.262	0.520 (0.764)	0.496	− 0.030 (0.192)	0.496	− 3.58 (0.92)	< 0.001
GM	− 0.948 (0.664)	0.153	0.120 (0.134)	0.370	− 0.383 (0.602)	0.525	0.115 (0.154)	0.525	− 3.55 (0.74)	< 0.001
WM	− 0.330 (0.584)	0.573	− 0.057 (0.080)	0.471	0.493 (0.384)	0.200	− 0.121 (0.094)	0.200	− 0.59 (0.44)	0.181
Ventricles	**1.944 (0.440)**	** < 0.001**	**0.099 (0.034)**	**0.004**	0.055 (0.091)	0.545	0.001 (0.024)	0.545	0.89 (0.19)	< 0.001
Frontal lobe	− 0.178 (0.420)	0.671	− 0.015 (0.061)	0.807	0.205 (0.279)	0.463	− 0.031 (0.070)	0.463	− 1.18 (0.34)	0.001
Frontal GM	− 0.187 (0.252)	0.458	0.004 (0.044)	0.932	0.008 (0.194)	0.966	0.005 (0.050)	0.966	− 0.85 (0.24)	< 0.001
Frontal WM	0.031 (0.264)	0.906	− 0.020 (0.033)	0.556	0.200 (0.161)	0.214	− 0.036 (0.039)	0.214	− 0.31 (0.18)	0.089
Temporal lobe	− 0.375 (0.247)	0.129	0.018 (0.037)	0.637	0.087 (0.162)	0.591	− 0.015 (0.042)	0.591	− 0.91 (0.21)	< 0.001
Temporal GM	− 0.202 (0.162)	0.213	0.008 (0.029)	0.784	− 0.001 (0.121)	0.993	0.004 (0.031)	0.993	− 0.70 (0.16)	< 0.001
Temporal WM	− 0.158 (0.146)	0.281	0.005 (0.020)	0.787	0.094 (0.093)	0.311	− 0.021 (0.023)	0.311	− 0.17 (0.11)	0.123
Parietal lobe	− 0.014 (0.238)	0.953	0.002 (0.033)	0.957	0.084 (0.159)	0.595	− 0.014 (0.039)	0.595	− 0.46 (0.18)	0.012
Parietal GM	− 0.033 (0.149)	0.824	0.027 (0.025)	0.287	− 0.033 (0.119)	0.778	0.022 (0.030)	0.778	− 0.46 (0.14)	0.001
Parietal WM	0.024 (0.139)	0.863	− 0.025 (0.018)	0.161	0.121 (0.090)	0.180	− 0.038 (0.022)	0.180	0.02 (0.10)	0.879
Occipital lobe	− 0.137 (0.199)	0.491	0.024 (0.027)	0.365	− 0.122 (0.137)	0.375	0.016 (0.033)	0.375	− 0.53 (0.15)	< 0.001
Occipital GM	− 0.082 (0.139)	0.557	0.045 (0.024)	0.059	− 0.168 (0.119)	0.158	0.039 (0.028)	0.158	− 0.59 (0.13)	< 0.001
Occipital WM	− 0.059 (0.091)	0.513	− 0.022 (0.012)	0.065	0.036 (0.059)	0.537	− 0.020 (0.014)	0.537	0.07 (0.07)	0.275
Hippocampus	− 0.003 (0.015)	0.840	− 0.000 (0.002)	0.803	− 0.000 (0.007)	0.968	− 0.000 (0.002)	0.968	− 0.03 (0.01)	0.002

*GM* gray matter, *WM* white matter.

All bolded values mean p-value < 0.01 (using false discovery rate for multiple comparison).

^a^Model (model 3) adjusted for ICV, the latest diagnostic status, baseline age, sex, race, years of education, height, fat mass, lean mass, number of multi-morbidities, CES-D, APOE e4 status and brisk walking. All β represent the change in brain volumes with 10% increase in baseline/change in cost to capacity ratio.

When examining the independent associations of energetic cost of slow walking and peak walking energy expenditure with brain volumes, in the fully adjusted models: (i) a 1 ml/kg/min higher baseline energetic cost of slow walking was associated with a 0.09 cm^3^ (SE = 0.03, p = 0.004) greater annual increase in ventricular volume (Supplementary Table 2, Column B), and (ii) a 1 ml/kg/min higher baseline peak walking energy expenditure was associated with a 0.67 cm^3^ (SE = 0.17, p < 0.001) smaller ventricular volume at baseline (Supplementary Table 3, Column A).

Our results largely remained robust with sensitivity analyses. When restricted to participants who never developed MCI or dementia over extended follow-up, a higher baseline cost-to-capacity ratio remained associated with a larger baseline ventricular volume (p < 0.001) and an annual increase in ventricular volume (p = 0.005) (Supplementary Table 4). Interaction terms with cost-to-capacity × subsequent normal/MCI/dementia status and cost-to-capacity × APOE status were not significant at the FDR-adjusted level (p < 0.01) and removed from the final models.

Further, we found some discrepancies when stratifying by baseline cost-to-capacity ratio: among those with baseline cost-to-capacity ratio ≤ 0.5 (good/modest energy utilization), a higher baseline cost-to-capacity ratio was only associated with an annual increase in ventricular volume (β = 0.16, SE = 0.08, p = 0.045) at the non-FDR adjusted level, but not the main baseline effect of brain volumes. Whereas, among those with a baseline cost-to-capacity ratio > 0.5 (poor energy utilization), a higher baseline cost-to-capacity ratio was only associated with a larger baseline ventricular volume (β = 2.34, SE = 0.91, p = 0.010) but not with annual changes in any brain volumes (Supplementary Table 5).

## Discussion

This prospective longitudinal study among middle-to-older-aged adults found that poorer energy utilization was associated with larger ventricular volume and steeper increase in ventricular volume over a mean 3.9 years of follow-up. However, no associations were found with other brain regions, and longitudinal change in energy utilization over time was not associated with brain structure. Notably, those with relatively efficient energy utilization at baseline, as indicated by a cost-to-capacity ratio ≤ 0.5, had a trending association between baseline cost-to-capacity ratio and subsequent annual increase in ventricular volume, whereas those with poor baseline energy utilization did not, hinting at a possible threshold effect. Collectively, these findings suggest that adverse changes in energy utilization may contribute to increases in ventricular volume early in the pathological process when energy utilization is still relatively efficient, warranting future follow-up in younger populations.

Ventricular enlargement provides an indirect measure of atrophy of central brain structures^24^. Although ventricular enlargement is seen with normal aging, it is also evident in a variety of neurodegenerative diseases, including Alzheimer’s disease^25^. In the current study, we surmise that the association between energy utilization and brain volumes limited to ventricular enlargement at baseline may be explained as follows: higher cardiorespiratory fitness has been associated with smaller ventricles^7,26^, and higher cardiorespiratory fitness also contributes to better energy utilization. Thus, it is plausible that energy utilization, defined by both cost and capacity, is related to ventricular enlargement. However, other brain-related diseases, such as Lewy body disease and vascular dementia, where gait dysfunction is common, may also be potential underlying contributors to the association between energy utilization and ventricular enlargement^27^. The majority of our impairments in our sample were Alzheimer’s disease and only a small number were dementia for other reasons (n = 19). As a result, we are unable to further disentangle the association regarding dementia subtypes.

Contrary to our hypothesis, changes in energy utilization were not related to annual changes in brain volumes. This may be due to the relatively older mean baseline age of the current sample (70 years old), or a potential threshold effect of energy utilization on brain atrophy. In our sample, change in cost-to-capacity ratio (0.04 ± 0.13) was minimal over the follow-up years, which could restrict our statistical power to detect significant associations. More importantly, the average cost-to-capacity ratio at baseline was 0.55 in this population, indicating that 55% of peak walking energy was used for ambulation. Previous work suggests that the cost-to-capacity ratio begins to increase around age 50, with an exponential increase each decade thereafter^8^. Thus, it is possible that once individuals exceed a cost-to-capacity ratio threshold of 40–50%, the effect of energy utilization on brain atrophy has already occurred, and subsequent changes in energy utilization have no further effect on brain volumes. We tested such possibility in the stratified analysis based on baseline energy utilization at a cost-to-capacity ratio of ≤ 0.50 vs > 0.50. We found that when baseline energy utilization was good/moderate (≤ 0.5), it was marginally associated with subsequent annual changes in ventricular volume; yet when baseline energy regulation was poor (> 0.50), there was no association with annual changes in brain volumes. Collectively, these results support our conjecture, but we cannot rule out the possibility of survival bias; those with less efficient energy utilization were less likely to return for follow-up visits, possibly attenuating longitudinal effects. Future studies with a longer follow-up time and more younger populations are warranted to disentangle these associations.

When examining the independent components of the cost ratio, we found that baseline energetic cost of slow walking was associated with an annual increase in ventricular enlargement, while baseline peak walking energy expenditure was associated with smaller ventricular volume at baseline only. These findings illustrate the differential utility of these energetic measures in understanding changes in brain structure with aging. Previous work from the BLSA examining the bidirectional association of time to walk 400 m, a validated measure of cardiorespiratory fitness^17^, with brain volumes also showed that fitness was not associated with future changes in ventricular volume, but smaller ventricular volume predicted future higher fitness^7^. The current study adds to this body of literature by illustrating that energetic cost of walking can be informative beyond measures of fitness, showing stronger associations with future brain changes, and adding to the growing body of literature linking the brain and aspects of mobility. Indeed, another recent study from the BLSA further showed that energetic cost of usual-paced walking was associated with accelerated annual increase in ventricular volume and decline in hippocampal volume^28^. Future research is needed to fully understand the association between the energy needed for walking at various speeds and brain volumes, especially among those less healthy than BLSA participants who may be more susceptible to dementia-related pathology.

To our knowledge, this is the first longitudinal study examining energy utilization—with a novel measure of energy cost and energy capacity—and brain volumes. However, the present study is not without limitations. First, the BLSA participants are highly educated, mostly Caucasian, and relatively healthy, thus findings may not generalize to other populations of older adults. Second, about half (49.6%) of the sample only contributed to the baseline data, and mid-life BLSA participants contribute less longitudinal data due to a longer interval between follow-up visits. Finally, the BLSA is observational, and although its prospective design allows for assessment of temporality between energy utilization and subsequent changes in brain volumes, we cannot rule out unmeasured confounding and reverse causality—that brain changes in early life may affect energy utilization in mid-to-late life.

In conclusion, less efficient energy utilization at baseline is associated with subsequent annual ventricle enlargement among middle-to-older-aged adults. Future longitudinal studies are needed to better characterize the value of energy utilization, which may be a potential early indicator of susceptibility to Alzheimer’s disease, in understanding changes in brain structure in younger and middle-aged adults.

## Supplementary Information

Supplementary Tables.

## Data Availability

Because of the sensitive nature of the data collected for this study, requests to access the data set from qualified researchers trained in human subject confidentiality protocols may be sent to the Intramural Research Program of the National Institute on Aging at https://blsa.nih.gov.
