# Supplementary material for: Longitudinal associations between energy utilization and brain volumes in cognitively normal middle aged and older adults
Source: Sci Rep. 2022 Apr 19;12:6472. doi: 10.1038/s41598-022-10421-7 (PMC9018828; doi:10.1038/s41598-022-10421-7)
Supplement: Supplementary file 1 — Supplementary Tables. [file 41598_2022_10421_MOESM1_ESM.docx]

**Longitudinal Associations between Energy Utilization and Brain Volumes in Cognitively Normal Middle Aged and Older Adults**

**Authors:**

Yujia (Susanna) Qiao, ScM^1*^; Amal A. Wanigatunga, PhD, MPH^1,2^; Yang An, MS^3^; Fangyu Liu, MHS^1^; Adam P. Spira, PhD^2,4,5^; Christos Davatzikos, PhD^6^; Qu Tian, PhD, MS^3^; Eleanor M. Simonsick, PhD^3^; Luigi Ferrucci, PhD^3^; Susan M. Resnick, PhD^3^; Jennifer A. Schrack, PhD^1,2^

**Affiliations:**

# 1) Department of Epidemiology, Johns Hopkins Bloomberg School of Public Health, Baltimore,

# Maryland, USA

# 2) Center on Aging and Health, Johns Hopkins University, Baltimore, Maryland, USA

# 3) Intramural Research Program, National Institute on Aging, Baltimore, Maryland USA

4) Department of Mental Health, Johns Hopkins Bloomberg School of Public Health, Baltimore, Maryland, USA

5) Department of Psychiatry and Behavioral Sciences, Johns Hopkins School of Medicine, Baltimore, Maryland, USA

6) Department of Radiology, University of Pennsylvania, Philadelphia, Pennsylvania, USA

## Appendices

**Supplement Table 1.** Baseline characteristics of 53 BLSA participants excluded from the main analyses due to missing covariates, by baseline cost-to-capacity ratio tertile

|  | **Overall**  **N=53** | **Highest Tertile**  **(0.59-1.0)**  **n=22** | **Middle Tertile**  **(0.48-0.59)**  **n=15** | **Lowest Tertile**  **(0.18-0.48)**  **n=16** | **P-trend** |
| --- | --- | --- | --- | --- | --- |
| Age (years) | 73.1 ± 9.5 | 76.9 ± 9.6 | 72.5 ± 9.0 | 68.5 ± 7.9 | 0.006 |
| Women | 26 (49.1) | 13 (59.1) | 6 (40.0) | 7 (43.8) | 0.459 |
| White | 38 (71.7) | 14 (63.6) | 10 (66.7) | 14 (87.5) | 0.239 |
| Education (years) | 17.5 ± 2.7 | 17.4 ± 3.1 | 18.5 ± 2.4 | 16.6 ± 2.4 | 0.499 |
| Height, in cm | 168.2 ± 8.3 | 165.9 ± 7.4 | 169.5 ± 10.1 | 170.2 ± 7.5 | 0.105 |
| Fat mass (kg) | 28.7 ± 12.6 | 34.8 ± 13.4 | 26.9 ± 11.7 | 19.5 ± 3.2 | 0.006 |
| Lean mass (kg) | 46.8 ± 8.7 | 45.7 ± 6.1 | 49.2 ± 11.4 | 46.0 ± 10.2 | 0.809 |
| Current smoker | 0 (0.0) | 0 (0.0) | 0 (0.0) | 0 (0.0) | . |
| Heavy drinker ^a^ | 8 (15.1) | 4 (18.2) | 2 (13.3) | 2 (12.5) | 0.868 |
| Having hypertension ^b^ | 30 (56.6) | 14 (63.6) | 13 (86.7) | 3 (18.8) | <0.001 |
| Having diabetes ^c^ | 9 (17.0) | 3 (13.6) | 4 (26.7) | 2 (12.5) | 0.496 |
| Have more than 2 comorbidities | 38 (71.7) | 15 (68.2) | 15 (100.0) | 8 (50.0) | 0.008 |
| CES-D | 5.8 ± 4.7 | 6.6 ± 5.8 | 6.1 ± 4.3 | 4.5 ± 3.5 | 0.249 |
| APOE e4 carriers | 12 (23.5) | 6 (27.3) | 1 (6.7) | 5 (35.7) | 0.157 |
| Intracranial volume (cm^3^) | 1410.56 ± 150.3 | 1357.8 ± 165.2 | 1421.8 ± 123.8 | 1469.1 ± 133.4 | 0.053 |
| Baseline cost-to-capacity ratio | 0.58 ± 0.16 | 0.74 ± 0.10 | 0.53 ± 0.04 | 0.40 ± 0.07 | <0.001 |

All reported in mean ± SD or frequency (%). Abbreviation: SD, standard deviation; BMI, body mass index.

^a^ Drinking more than 7 alcoholic drinks/week.

^b^ Systolic blood pressure ≥ 130 mm Hg and or diastolic blood pressure ≥ 80 mm Hg, or a history of diagnosis plus treatment with antihypertensive medications.

^c^ Fasting glucose ≥ 126 mg/dL, or a history of a diagnosis plus treatment with oral antidiabetic drugs or insulin.

**Supplement Table 2.** Longitudinal mixed models of the associations between energetic cost of slow walking (ml/kg/min) and brain volumes (cm^3^) among 703 BLSA participants ^a^

|  | **Baseline** **energetic cost of slow walking**  **(A)** | | **Baseline** **energetic cost of slow walking *time**  **(B)** | | **Change in energetic cost of slow walking**  **(C)** | | **Change in energetic cost of slow walking*time**  **(D)** | | **Time**  **(E)** | |
| --- | --- | --- | --- | --- | --- | --- | --- | --- | --- | --- |
|  | β (SE) | p-value | β (SE) | p-value | β (SE) | p-value | β (SE) | p-value | β (SE) | p-value |
| Total brain | -0.515 (0.855) | 0.547 | 0.209 (0.149) | 0.161 | 0.509 (0.714) | 0.476 | -0.042 (0.183) | 0.476 | -4.37 (1.30) | 0.001 |
| GM | -0.131 (0.560) | 0.815 | 0.090 (0.120) | 0.454 | 0.334 (0.563) | 0.554 | -0.029 (0.147) | 0.554 | -3.66 (1.05) | 0.001 |
| WM | -0.678 (0.490) | 0.166 | -0.005 (0.071) | 0.949 | 0.250 (0.361) | 0.489 | -0.060 (0.090) | 0.489 | -0.87 (0.62) | 0.164 |
| Ventricles | 0.611 (0.378) | 0.106 | **0.089 (0.031)** | **0.004** | -0.037 (0.084) | 0.655 | 0.025 (0.023) | 0.655 | 0.67 (0.27) | 0.015 |
| Frontal lobe | -0.294 (0.352) | 0.405 | 0.020 (0.055) | 0.711 | 0.310 (0.262) | 0.237 | -0.055 (0.067) | 0.237 | -1.42 (0.48) | 0.003 |
| Frontal GM | -0.075 (0.211) | 0.722 | 0.018 (0.039) | 0.651 | 0.205 (0.182) | 0.261 | -0.038 (0.047) | 0.261 | -0.97 (0.34) | 0.005 |
| Frontal WM | -0.228 (0.222) | 0.304 | 0.003 (0.030) | 0.928 | 0.108 (0.151) | 0.474 | -0.013 (0.038) | 0.474 | -0.43 (0.26) | 0.095 |
| Temporal lobe | -0.035 (0.208) | 0.865 | 0.000 (0.034) | 0.999 | 0.134 (0.152) | 0.376 | -0.028 (0.040) | 0.376 | -0.82 (0.29) | 0.006 |
| Temporal GM | 0.108 (0.136) | 0.429 | -0.014 (0.026) | 0.595 | 0.132 (0.113) | 0.242 | -0.025 (0.030) | 0.242 | -0.54 (0.23) | 0.017 |
| Temporal WM | -0.135 (0.123) | 0.270 | 0.008 (0.018) | 0.640 | 0.027 (0.087) | 0.760 | -0.009 (0.022) | 0.760 | -0.22 (0.16) | 0.167 |
| Parietal lobe | -0.080 (0.200) | 0.689 | 0.024 (0.030) | 0.415 | 0.199 (0.149) | 0.183 | -0.025 (0.037) | 0.183 | -0.66 (0.26) | 0.011 |
| Parietal GM | 0.025 (0.125) | 0.841 | 0.025 (0.023) | 0.265 | 0.128 (0.112) | 0.252 | -0.010 (0.028) | 0.252 | -0.53 (0.20) | 0.007 |
| Parietal WM | -0.100 (0.117) | 0.391 | 0.000 (0.016) | 0.994 | 0.085 (0.085) | 0.314 | -0.017 (0.021) | 0.314 | -0.13 (0.14) | 0.356 |
| Occipital lobe | 0.042 (0.167) | 0.800 | 0.009 (0.024) | 0.693 | -0.113 (0.130) | 0.384 | 0.026 (0.031) | 0.384 | -0.49 (0.21) | 0.020 |
| Occipital GM | 0.106 (0.117) | 0.366 | 0.027 (0.021) | 0.201 | -0.084 (0.112) | 0.456 | 0.037 (0.027) | 0.456 | -0.58 (0.18) | 0.002 |
| Occipital WM | -0.063 (0.076) | 0.406 | -0.017 (0.011) | 0.104 | -0.013 (0.055) | 0.808 | -0.013 (0.014) | 0.808 | 0.10 (0.09) | 0.302 |
| Hippocampus | -0.007 (0.013) | 0.601 | -0.000 (0.002) | 0.871 | 0.007 (0.006) | 0.238 | -0.002 (0.002) | 0.238 | -0.03 (0.02) | 0.026 |

Abbreviation: GM, gray matter; WM, white matter.

All bolded values mean p-value< 0.01 (using false discovery rate for multiple comparison).

^a^ Model (model 3) adjusted for ICV, the latest diagnostic status, years of follow-up, baseline age, sex, race, years of education, height, fat mass, lean mass, number of multi-morbidities, CES-D, APOE e4 status, brisk walking, and time-varying peak walking energy expenditure. All β represent the change in brain volumes with 1 ml/kg/min increase in baseline/change in energetic cost of slow walking.

**Supplement Table 3.** Longitudinal mixed models of the associations between peak walking energy expenditure (ml/kg/min) and brain volumes (cm^3^) among 703 BLSA participants ^a^

|  | **Baseline peak walking energy expenditure**  **(A)** | | **Baseline peak walking energy expenditure*time**  **(B)** | | **Change in peak walking energy expenditure**  **(C)** | | **Change in peak walking energy expenditure*time**  **(D)** | | **Time**  **(E)** | |
| --- | --- | --- | --- | --- | --- | --- | --- | --- | --- | --- |
|  | β (SE) | p-value | β (SE) | p-value | β (SE) | p-value | β (SE) | p-value | β (SE) | p-value |
| Total brain | -0.200 (0.405) | 0.621 | -0.014 (0.059) | 0.816 | 0.024 (0.352) | 0.945 | 0.010 (0.086) | 0.945 | -2.28 (1.01) | 0.023 |
| GM | 0.570 (0.265) | 0.031 | -0.015 (0.047) | 0.747 | 0.338 (0.278) | 0.225 | -0.052 (0.069) | 0.225 | -2.61 (0.81) | 0.001 |
| WM | -0.145 (0.231) | 0.530 | 0.014 (0.028) | 0.611 | -0.146 (0.176) | 0.406 | 0.045 (0.042) | 0.406 | -1.10 (0.47) | 0.020 |
| Ventricles | **-0.667 (0.169)** | **<0.001** | -0.023 (0.012) | 0.068 | -0.038 (0.042) | 0.361 | 0.006 (0.011) | 0.361 | 1.80 (0.22) | <0.001 |
| Frontal lobe | 0.009 (0.167) | 0.957 | 0.007 (0.022) | 0.752 | 0.049 (0.129) | 0.701 | -0.009 (0.032) | 0.701 | -1.36 (0.37) | <0.001 |
| Frontal GM | 0.098 (0.100) | 0.327 | 0.002 (0.015) | 0.878 | 0.103 (0.090) | 0.250 | -0.020 (0.022) | 0.250 | -0.86 (0.26) | 0.001 |
| Frontal WM | -0.100 (0.104) | 0.339 | 0.004 (0.012) | 0.729 | -0.061 (0.074) | 0.405 | 0.014 (0.018) | 0.405 | -0.47 (0.20) | 0.018 |
| Temporal lobe | 0.166 (0.098) | 0.090 | -0.004 (0.013) | 0.791 | 0.022 (0.074) | 0.764 | 0.001 (0.019) | 0.764 | -0.75 (0.23) | 0.001 |
| Temporal GM | **0.165 (0.064)** | **0.010** | -0.005 (0.010) | 0.636 | 0.059 (0.056) | 0.291 | -0.009 (0.014) | 0.291 | -0.57 (0.17) | 0.001 |
| Temporal WM | -0.002 (0.058) | 0.968 | 0.001 (0.007) | 0.909 | -0.034 (0.043) | 0.426 | 0.009 (0.010) | 0.426 | -0.15 (0.12) | 0.209 |
| Parietal lobe | -0.045 (0.094) | 0.635 | 0.006 (0.012) | 0.618 | 0.039 (0.073) | 0.591 | -0.003 (0.018) | 0.591 | -0.54 (0.20) | 0.007 |
| Parietal GM | 0.014 (0.059) | 0.819 | -0.001 (0.009) | 0.902 | 0.078 (0.055) | 0.154 | -0.017 (0.013) | 0.154 | -0.30 (0.15) | 0.048 |
| Parietal WM | -0.057 (0.055) | 0.304 | 0.007 (0.006) | 0.258 | -0.034 (0.041) | 0.407 | 0.014 (0.010) | 0.407 | -0.23 (0.11) | 0.031 |
| Occipital lobe | 0.113 (0.079) | 0.151 | -0.001 (0.009) | 0.905 | 0.021 (0.063) | 0.732 | 0.012 (0.015) | 0.732 | -0.35 (0.16) | 0.027 |
| Occipital GM | 0.116 (0.055) | 0.035 | -0.005 (0.008) | 0.537 | 0.043 (0.055) | 0.435 | 0.003 (0.013) | 0.435 | -0.24 (0.14) | 0.085 |
| Occipital WM | -0.001 (0.036) | 0.968 | 0.004 (0.004) | 0.324 | -0.018 (0.027) | 0.505 | 0.008 (0.006) | 0.505 | -0.11 (0.07) | 0.109 |
| Hippocampus | -0.000 (0.006) | 0.957 | 0.001 (0.001) | 0.395 | 0.005 (0.003) | 0.090 | -0.001 (0.001) | 0.090 | -0.05 (0.01) | <0.001 |

Abbreviation: GM, gray matter; WM, white matter.

All bolded values mean p-value< 0.01 (using false discovery rate for multiple comparison).

^a^ Model (model 3) adjusted for ICV, the latest diagnostic status, years of follow-up, baseline age, sex, race, years of education, height, fat mass, lean mass, number of multi-morbidities, CES-D, APOE e4 status, brisk walking, and time-varying energetic cost of slow walking. All β represent the change in brain volumes with 1 ml/kg/min increase in baseline/change in peak walking energy expenditure.

**Supplement Table 4.** Longitudinal mixed models of the associations between energy utilization and brain volumes among 619 BLSA sub-population who did not develop MCI or dementia over extended follow-up ^a^

|  | **Baseline cost-to-capacity ratio**  **(A)** | | **Baseline cost-to-capacity ratio *time**  **(B)** | | **Change in cost-to-capacity ratio**  **(C)** | | **Change in cost-to-capacity ratio *time**  **(D)** | | **Time**  **(E)** | |
| --- | --- | --- | --- | --- | --- | --- | --- | --- | --- | --- |
|  | β (SE) | p-value | β (SE) | p-value | β (SE) | p-value | β (SE) | p-value | β (SE) | p-value |
| Total brain | -0.270 (1.103) | 0.807 | 0.080 (0.176) | 0.650 | 0.680 (0.858) | 0.429 | -0.257 (0.217) | 0.429 | -2.99 (0.96) | 0.002 |
| GM | -0.883 (0.715) | 0.217 | 0.041 (0.140) | 0.770 | -0.255 (0.671) | 0.704 | -0.039 (0.172) | 0.704 | -3.02 (0.77) | <0.001 |
| WM | -0.520 (0.635) | 0.414 | -0.088 (0.083) | 0.287 | 0.495 (0.443) | 0.264 | -0.145 (0.107) | 0.264 | -0.45 (0.45) | 0.319 |
| Ventricles | **1.672 (0.416)** | **<0.001** | **0.094 (0.033)** | **0.005** | 0.041 (0.097) | 0.670 | -0.006 (0.025) | 0.670 | 0.78 (0.18) | <0.001 |
| Frontal lobe | -0.311 (0.453) | 0.493 | -0.033 (0.066) | 0.612 | 0.274 (0.321) | 0.392 | -0.102 (0.081) | 0.392 | -1.06 (0.36) | 0.003 |
| Frontal GM | -0.181 (0.271) | 0.504 | -0.012 (0.047) | 0.791 | 0.025 (0.222) | 0.912 | -0.036 (0.057) | 0.912 | -0.74 (0.26) | 0.004 |
| Frontal WM | -0.102 (0.287) | 0.723 | -0.024 (0.035) | 0.495 | 0.247 (0.187) | 0.185 | -0.062 (0.045) | 0.185 | -0.30 (0.19) | 0.121 |
| Temporal lobe | -0.416 (0.261) | 0.112 | -0.011 (0.037) | 0.766 | 0.009 (0.174) | 0.959 | -0.025 (0.044) | 0.959 | -0.72 (0.20) | <0.001 |
| Temporal GM | -0.187 (0.171) | 0.274 | -0.005 (0.028) | 0.847 | -0.033 (0.133) | 0.805 | -0.006 (0.034) | 0.805 | -0.58 (0.16) | <0.001 |
| Temporal WM | -0.216 (0.157) | 0.169 | -0.011 (0.019) | 0.551 | 0.035 (0.101) | 0.733 | -0.017 (0.025) | 0.733 | -0.10 (0.11) | 0.364 |
| Parietal lobe | -0.033 (0.257) | 0.897 | -0.009 (0.036) | 0.798 | 0.135 (0.182) | 0.460 | -0.047 (0.045) | 0.460 | -0.39 (0.19) | 0.043 |
| Parietal GM | -0.031 (0.161) | 0.846 | 0.018 (0.027) | 0.494 | -0.027 (0.136) | 0.841 | 0.000 (0.034) | 0.841 | -0.40 (0.15) | 0.006 |
| Parietal WM | 0.005 (0.151) | 0.975 | -0.028 (0.019) | 0.136 | 0.164 (0.103) | 0.111 | -0.048 (0.024) | 0.111 | 0.02 (0.10) | 0.862 |
| Occipital lobe | -0.037 (0.216) | 0.866 | 0.005 (0.027) | 0.863 | -0.113 (0.149) | 0.448 | -0.007 (0.035) | 0.448 | -0.40 (0.15) | 0.006 |
| Occipital GM | -0.035 (0.150) | 0.818 | 0.028 (0.024) | 0.236 | -0.152 (0.128) | 0.232 | 0.013 (0.031) | 0.232 | -0.48 (0.13) | <0.001 |
| Occipital WM | -0.007 (0.099) | 0.940 | -0.025 (0.012) | 0.047 | 0.017 (0.067) | 0.798 | -0.015 (0.016) | 0.798 | 0.09 (0.07) | 0.202 |
| Hippocampus | -0.006 (0.016) | 0.722 | -0.003 (0.002) | 0.147 | -0.002 (0.007) | 0.778 | -0.000 (0.002) | 0.778 | -0.02 (0.01) | 0.050 |

Abbreviation: GM, gray matter; WM, white matter; MCI, mild cognitive impairment.

All bolded values mean P < 0.01 (using false discovery rate for multiple comparison).

^a^ Model adjusted for ICV, years of follow-up, baseline age, sex, race, years of education, height, fat mass, lean mass, number of multi-morbidities, CES-D, APOE e4 status and brisk walking.

**Supplement Table 5.** Longitudinal mixed models of the associations between energy utilization and brain volumes among 703 BLSA participants, by baseline cost-to-capacity ratio (≤ 0.5 vs. > 0.5) ^a^

| **(1)** **Baseline cost-to-capacity ratio ≤ 0.5 (n=301)** | | | | | | | | | | |
| --- | --- | --- | --- | --- | --- | --- | --- | --- | --- | --- |
|  | **Baseline cost-to-capacity ratio**  **(A)** | | **Baseline cost-to-capacity ratio *time**  **(B)** | | **Change in cost-to-capacity ratio**  **(C)** | | **Change in cost-to-capacity ratio *time**  **(D)** | | **Time**  **(E)** | |
|  | β (SE) | p-value | β (SE) | p-value | β (SE) | p-value | β (SE) | p-value | β (SE) | p-value |
| Total brain | -2.367 (2.445) | 0.333 | 0.341 (0.425) | 0.423 | -1.041 (1.144) | 0.363 | 0.093 (0.258) | 0.363 | -3.86 (1.85) | 0.037 |
| GM | -0.864 (1.689) | 0.609 | -0.002 (0.329) | 0.996 | -1.553 (0.885) | 0.080 | 0.174 (0.201) | 0.080 | -2.78 (1.43) | 0.052 |
| WM | -1.396 (1.442) | 0.333 | 0.150 (0.193) | 0.436 | 0.076 (0.588) | 0.897 | -0.087 (0.125) | 0.897 | -1.34 (0.84) | 0.114 |
| Ventricle | -0.706 (0.812) | 0.385 | **0.160 (0.080)** | **0.045** | -0.043 (0.117) | 0.715 | 0.041 (0.029) | 0.715 | 0.62 (0.34) | 0.063 |
| Frontal lobe | -0.582 (1.023) | 0.570 | 0.050 (0.161) | 0.756 | -0.414 (0.429) | 0.335 | 0.056 (0.097) | 0.335 | -1.34 (0.70) | 0.055 |
| Frontal GM | -0.324 (0.640) | 0.613 | 0.001 (0.112) | 0.995 | -0.423 (0.289) | 0.143 | 0.055 (0.066) | 0.143 | -0.78 (0.48) | 0.110 |
| Frontal WM | -0.287 (0.653) | 0.661 | 0.045 (0.083) | 0.587 | 0.016 (0.248) | 0.948 | -0.005 (0.053) | 0.948 | -0.54 (0.36) | 0.137 |
| Temporal lobe | -0.616 (0.622) | 0.322 | 0.068 (0.093) | 0.465 | -0.164 (0.231) | 0.479 | -0.028 (0.054) | 0.479 | -1.03 (0.40) | 0.010 |
| Temporal GM | -0.306 (0.419) | 0.465 | 0.010 (0.070) | 0.891 | -0.162 (0.171) | 0.343 | -0.006 (0.040) | 0.343 | -0.65 (0.30) | 0.033 |
| Temporal WM | -0.309 (0.362) | 0.394 | 0.063 (0.044) | 0.148 | 0.010 (0.134) | 0.939 | -0.029 (0.028) | 0.939 | -0.38 (0.19) | 0.044 |
| Parietal lobe | -0.416 (0.586) | 0.478 | 0.002 (0.082) | 0.985 | -0.266 (0.238) | 0.263 | 0.031 (0.052) | 0.263 | -0.42 (0.36) | 0.244 |
| Parietal GM | -0.097 (0.382) | 0.799 | -0.004 (0.061) | 0.954 | -0.269 (0.170) | 0.114 | 0.047 (0.038) | 0.114 | -0.31 (0.27) | 0.239 |
| Parietal WM | -0.333 (0.341) | 0.330 | 0.006 (0.045) | 0.887 | 0.009 (0.138) | 0.950 | -0.018 (0.029) | 0.950 | -0.11 (0.20) | 0.583 |
| Occipital lobe | 0.296 (0.509) | 0.561 | -0.001 (0.066) | 0.990 | -0.383 (0.206) | 0.063 | 0.031 (0.043) | 0.063 | -0.36 (0.29) | 0.211 |
| Occipital GM | 0.329 (0.360) | 0.361 | -0.018 (0.055) | 0.750 | -0.381 (0.170) | 0.025 | 0.051 (0.036) | 0.025 | -0.29 (0.24) | 0.236 |
| Occipital WM | -0.050 (0.233) | 0.832 | 0.017 (0.028) | 0.556 | 0.012 (0.088) | 0.888 | -0.020 (0.019) | 0.888 | -0.08 (0.12) | 0.527 |
| Hippocampus | -0.004 (0.036) | 0.903 | -0.003 (0.004) | 0.531 | 0.003 (0.009) | 0.763 | -0.002 (0.002) | 0.763 | -0.02 (0.02) | 0.253 |

| **(2)** **Baseline cost-to-capacity ratio > 0.5 (n=402)** | | | | | | | | | | |
| --- | --- | --- | --- | --- | --- | --- | --- | --- | --- | --- |
|  | **Baseline cost-to-capacity ratio**  **(A)** | | **Baseline cost-to-capacity ratio *time**  **(B)** | | **Change in cost-to-capacity ratio**  **(C)** | | **Change in cost-to-capacity ratio *time**  **(D)** | | **Time**  **(E)** | |
|  | β (SE) | p-value | β (SE) | p-value | β (SE) | p-value | β (SE) | p-value | β (SE) | p-value |
| Total brain | 2.461 (1.873) | 0.189 | 0.284 (0.306) | 0.354 | 1.432 (1.098) | 0.193 | -0.079 (0.299) | 0.193 | -4.42 (1.94) | 0.023 |
| GM | -0.112 (1.168) | 0.924 | 0.285 (0.254) | 0.262 | 0.437 (0.885) | 0.622 | 0.071 (0.245) | 0.622 | -4.71 (1.61) | 0.004 |
| WM | 1.165 (1.049) | 0.267 | -0.092 (0.151) | 0.543 | 0.773 (0.535) | 0.149 | -0.171 (0.146) | 0.149 | -0.47 (0.96) | 0.626 |
| Ventricle | **2.342 (0.907)** | **0.010** | 0.095 (0.067) | 0.158 | 0.101 (0.140) | 0.468 | -0.022 (0.040) | 0.468 | 0.90 (0.43) | 0.038 |
| Frontal lobe | 1.069 (0.754) | 0.157 | 0.018 (0.107) | 0.865 | 0.590 (0.389) | 0.130 | -0.090 (0.105) | 0.130 | -1.45 (0.68) | 0.033 |
| Frontal GM | 0.230 (0.436) | 0.599 | 0.019 (0.079) | 0.807 | 0.281 (0.281) | 0.317 | -0.030 (0.077) | 0.317 | -0.97 (0.50) | 0.056 |
| Frontal WM | 0.870 (0.474) | 0.067 | -0.011 (0.061) | 0.850 | 0.335 (0.222) | 0.132 | -0.071 (0.060) | 0.132 | -0.40 (0.38) | 0.294 |
| Temporal lobe | -0.538 (0.437) | 0.218 | 0.030 (0.071) | 0.668 | 0.159 (0.239) | 0.507 | 0.029 (0.066) | 0.507 | -1.06 (0.45) | 0.019 |
| Temporal GM | -0.319 (0.280) | 0.254 | 0.027 (0.055) | 0.631 | 0.045 (0.181) | 0.804 | 0.034 (0.051) | 0.804 | -0.86 (0.35) | 0.014 |
| Temporal WM | -0.186 (0.261) | 0.476 | 0.001 (0.042) | 0.990 | 0.113 (0.133) | 0.396 | -0.009 (0.037) | 0.396 | -0.16 (0.27) | 0.542 |
| Parietal lobe | 0.800 (0.425) | 0.061 | 0.029 (0.060) | 0.634 | 0.387 (0.227) | 0.088 | -0.068 (0.060) | 0.088 | -0.67 (0.38) | 0.083 |
| Parietal GM | 0.366 (0.260) | 0.160 | 0.062 (0.047) | 0.188 | 0.165 (0.177) | 0.353 | -0.006 (0.047) | 0.353 | -0.70 (0.30) | 0.019 |
| Parietal WM | 0.462 (0.248) | 0.063 | -0.041 (0.035) | 0.241 | 0.218 (0.126) | 0.084 | -0.064 (0.034) | 0.084 | 0.10 (0.22) | 0.665 |
| Occipital lobe | -0.040 (0.346) | 0.907 | 0.081 (0.050) | 0.106 | 0.090 (0.198) | 0.648 | 0.006 (0.051) | 0.648 | -0.92 (0.32) | 0.004 |
| Occipital GM | -0.112 (0.240) | 0.641 | 0.111 (0.047) | 0.019 | 0.014 (0.180) | 0.936 | 0.032 (0.048) | 0.936 | -1.03 (0.30) | 0.001 |
| Occipital WM | 0.085 (0.158) | 0.590 | -0.036 (0.024) | 0.137 | 0.055 (0.083) | 0.510 | -0.024 (0.023) | 0.510 | 0.14 (0.15) | 0.348 |
| Hippocampus | -0.027 (0.028) | 0.344 | 0.001 (0.004) | 0.843 | -0.006 (0.011) | 0.599 | 0.002 (0.003) | 0.599 | -0.04 (0.02) | 0.091 |

Abbreviation: GM, gray matter; WM, white matter.

All bolded values mean p< 0.05.

^a^ Model (model 3) adjusted for ICV, the latest diagnostic status, years of follow-up, baseline age, sex, race, years of education, height, fat mass, lean mass, number of multi-morbidities, CES-D, APOE e4 status and brisk walking.
